# Pituitary adenylate cyclase-activating polypeptide (PACAP) in the paraventricular nucleus of the thalamus: Influence on binge-type eating in male and female mice

**DOI:** 10.21203/rs.3.rs-4145128/v1

**Published:** 2024-04-02

**Authors:** Genevieve R. Curtis, Brody A. Carpenter, Breanne E. Pirino, Annie Hawks, George Li, Jessica R. Barson

**Affiliations:** 1 Department of Neurobiology and Anatomy, Drexel University College of Medicine, Philadelphia, P.A. 19129

## Abstract

Binge eating disorder, characterized by the overconsumption of food in a discrete time period, is the most common eating disorder in the United States, but its neurological basis is not fully understood. The paraventricular nucleus of the thalamus (PVT) is a limbic brain region implicated in eating, and the anorexigenic neuropeptide, pituitary adenylate cyclase-activating polypeptide (PACAP), is densely expressed in the PVT. This study sought to examine the possible involvement of PACAP in the PVT in binge-type eating. First, a model of binge-type eating was established in mice. Male and female C57BL/6J mice were given limited access to Milk Chocolate Ensure Plus^®^ or had access only to chow and water. Under this model, while males and females both engaged in binge-type eating with Ensure, females engaged in this behavior to a greater degree than males. Next, the role of PACAP in the PVT was defined in relation to binge-type eating. Using quantitative real-time PCR, females were found to have higher baseline levels of PVT PACAP mRNA than males, but only males showed an increase in levels of PACAP after a history of binge-type eating, and only males showed a reduction in levels of PACAP immediately prior to a binge session. Using chemogenetics in PACAP-Cre transgenic mice on a C57BL/6J background, activation of PVT PACAP^+^ cells with a Cre-dependent Gq-DREADD was found to reduce binge-type eating, significantly in male but not female mice. These results indicate that PVT PACAP is involved in binge-type eating in a sex-dependent manner, with a decrease in PVT PACAP levels preceding binge-type eating in male mice, and enhanced PVT PACAP^+^ cell activity suppressing binge-type eating in male mice. Together, these results suggest that the PACAP system could be targeted in specific patient populations to help treat binge eating disorder.

## Introduction

Binge eating disorder is the most common eating disorder in the United States [1]. While this disorder affects more women than men, it is also the eating disorder with the most equal representation between women and men [1,2]. Clinically, binge eating disorder is characterized by the consumption of an unusually large amount of food in a short period of time, despite the absence of hunger and without compensatory behaviors [3]. Pre-clinically, binge-type eating is defined by: (1) the consumption of a larger than normal amount of food compared to a control group during the same period of time; and / or (2) escalated intake across sessions [4]. Many animal models induce binge-type eating by limiting access to a palatable food, typically one high in fat and / or carbohydrates [5–12]. These models do not always induce weight gain [6,8–11]. While animal models of binge-type eating have historically predominantly used rats, recent studies have begun to use mice (e.g. [5,13,14]). In both rats and mice, limited published work suggests that females binge at higher levels than males [15–17].

One brain region that may be involved in binge-type eating is the paraventricular nucleus of the thalamus (PVT), a midline limbic structure [18–22]. While there is evidence for the involvement of the PVT in feeding behavior, the direction of this involvement is unclear. For example, in rats, activation of the PVT and its projections has been found to increase sucrose seeking and intake [23–25], while inactivation of the PVT has also been found to increase sucrose seeking [26] and chow intake [27] and, in mice, stimulation of inhibitory GABA-ergic inputs into the PVT induces rapid consumption of high-fat, sweet, and standard foods [28]. The discrepancies in these findings may be due to targeting of distinct neuronal populations in the PVT.

One of the most highly expressed neuropeptides in the PVT is pituitary adenylate cyclase-activating polypeptide (PACAP) [29–31]. With preproPACAP independently processed into two peptide isoforms, PACAP-38 and PACAP-27 [32], we have found that less common isoform, PACAP-27, is the predominant isoform in the PVT, highly expressed in both the rat and mouse, and that expression of PACAP is greater in the posterior half of the PVT [31,33]. Both isoforms of PACAP have been found with systemic injection to inhibit intake of standard chow [34,35], and PACAP-38 has been found with injection into the ventral tegmental area to inhibit binge-type eating [36].

The purpose of this study was to examine the involvement of PVT PACAP in binge-type eating. We hypothesized that females would engage in binge-type eating to a greater degree than males, that endogenous levels of PACAP mRNA would be reduced in the PVT prior to binge-type eating, and that enhanced activity in these PVT PACAP^+^ cells would attenuate binge-type eating.

## Materials and Methods

### Subjects.

Adult male and female heterozygous PACAP-Cre transgenic (Adcyap1-2A-Cre) mice or wild-type littermate mice on a C57BL/6J background (*N* = 70, 35 male, 35 female, 23 wild-type, 47 PACAP-Cre; bred in-house from mice originally purchased from Jackson Laboratory, Bar Harbor, ME, USA) were individually housed starting at 7 – 11 weeks old, one week before the start of testing, in an AAALAC accredited facility, on a 12-hour reversed light/dark cycle, with lights off at 0600 h. All mice received *ad libitum* water and chow (Laboratory Rodent Diet 5001, Lab Diet, St. Louis, MO, USA) throughout the study. Experiments were approved by the Institutional Animal Care and Use Committee of Drexel University College of Medicine and complied with the ARRIVE guidelines, carried out in accordance with the National Institutes of Health Guide for the Care and Use of Laboratory Animals [37].

### Experimental Protocols

**Figure 1.**
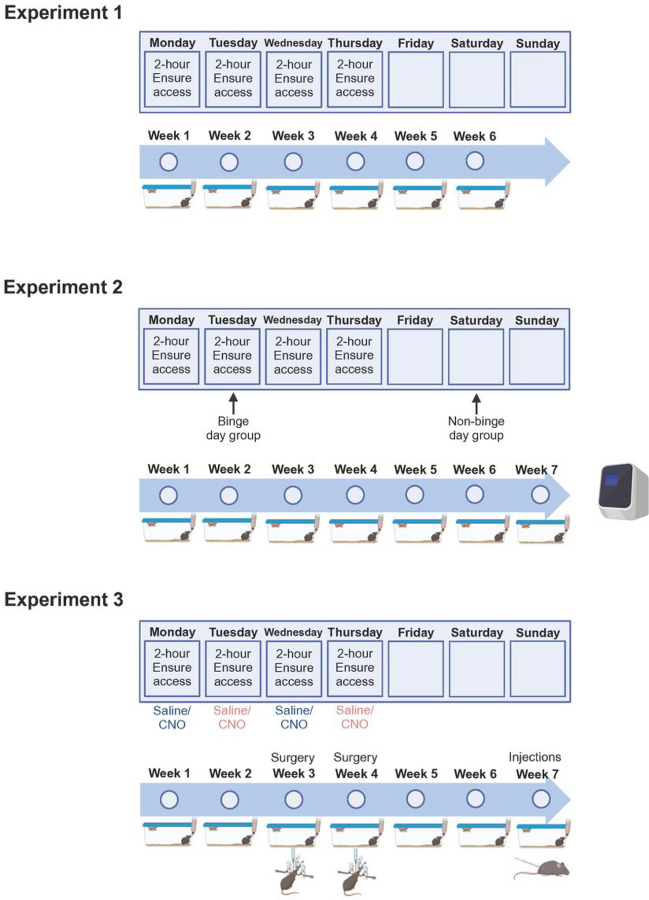
Experimental timelines. In *Experiment 1*, mice were given 2-hour access to Ensure (or no access), 4 days per week for 6 weeks, and intake and body weight were measured. In *Experiment 2*, mice from Experiment 1 were sacrificed during the 7^th^ week of the binge-type eating paradigm, immediately prior to the start of the binge session (binge day group or Control) or at the equivalent time of a day without Ensure access (non-binge day group or Control), and their gene expression in the paraventricular nucleus of the thalamus (PVT) was examined using quantitative real-time polymerase chain reaction. In *Experiment 3*, mice underwent the binge-type eating paradigm, were injected in the PVT with a Cre-dependent excitatory DREADD or control virus during the 3^rd^ or 4^th^ week of the paradigm, and then were injected with clozapine n-oxide (CNO) or saline vehicle during the 7^th^ week of the paradigm, 30 minutes prior to their binge eating session. After 48 hours, they were injected with the other drug, 30 minutes prior to their binge eating session. Intake was recorded after the injections.

See Figure 1 for experimental timelines.

### Experiment 1. Model of binge-type eating.

Male and female mice (of both wild-type and PACAP-Cre genotypes) were given 2-hour access to Ensure (see below), 4 days per week for 6 weeks (Binge group; *n* = 12/sex) or were not given access to Ensure (Control group; *n* = 12 females, *n* = 8 males).

### Experiment 2. PACAP gene expression in the PVT relative to binge-type eating.

Male and female mice from the Binge group (*n* = 6/sex/group) in Experiment 1 were sacrificed following the 6^th^ week of the binge-type eating paradigm, immediately prior to the start of the binge session (binge day group), or at the equivalent time of a day without Ensure access (non-binge day group). A subset of the male and female Control mice (*n* = 6/sex) from Experiment 1 were sacrificed at the same time of day and days of the week as the binge and non-binge day groups. Sacrifice was via rapid decapitation. mRNA was examined using quantitative real-time polymerase chain reaction (qRT-PCR).

### Experiment 3: Effects of PACAP^+^ cell activity in the PVT on binge-type eating.

Male and female PACAP-Cre transgenic mice (*n* = 15 males, 11 females) underwent the binge-type eating paradigm, and received injections into the PVT of a Cre-dependent excitatory DREADD (*n* = 14) or control virus (*n* = 12) during the 3^rd^ or 4^th^ week of the paradigm. Following 6 total weeks of binge-type eating, all mice received intraperitoneal (i.p.) injections of 5 mg/kg clozapine n-oxide (CNO; HelloBio, Princeton, NJ, USA) or saline vehicle (~0.14 mL; Baxter International Inc., Deerfield, IL, USA), 30 minutes prior to their binge eating session, and subsequent intake was recorded. These injections were made within-subject, with 48 hours between injections. One week later, to ensure that Cre was co-expressed with PACAP peptide, and that injection of CNO resulted in excitation of these Cre^+^ cells, a sub-set of the mice was injected between-subject with CNO or saline and sacrificed 120 minutes later. Immunohistochemistry was then used to detect Cre, PACAP, and c-Fos.

#### Binge-type eating paradigm.

Starting 4 hours after lights off, *ad libitum* fed mice were given two-hour access to Milk Chocolate Ensure Plus^®^ (Abbott Nutrition, East Windsor, NJ, USA) in 16-ounce bottles with non-drip sipper tubes. This was repeated for 4 days per week (Monday - Thursday). The Ensure contained 1.32 kcal/g and was comprised of 57% carbohydrate (23% sucrose), 28% fat, and 15% protein. The chow diet (Laboratory Rodent Diet 5001) contained 3.36 kcal/g and was comprised of 58% carbohydrate (8% sucrose), 13% fat, and 29% protein. Chow, water, and Ensure, when present, were weighed before and after each session. Body weight was measured at least once each week. A 4500 Hz tone was played immediately before delivery of Ensure to each mouse, to cue the beginning of the binge eating session.

#### Quantitative real time PCR.

For details of PVT dissection, RNA extraction, cDNA reverse transcription, primer sequences, and qRT-PCR, see [33]. Briefly, whole PVT was dissected immediately after sacrifice and stored at −20 °C in RNA*later* (Qiagen Inc., Valenia, CA, USA). Total RNA from each brain sample was then extracted, and the yield was quantified, with A260/A280 ratios between 1.85 and 2.27, indicating high purity. Primers for qRT-PCR were PACAP and cyclophilin-A (the housekeeping gene), with gene expression of PACAP quantified using the relative quantification method (ΔΔC_T_).

#### Stereotaxic surgery.

Mice were injected with 0.25 μL of Cre-dependent excitatory DREADD (pAAV8-hSyn-DIO-hM3D(G_q_)-mCherry, Addgene, Watertown, MA, USA) or control virus (pAAV8-hSyn-DIO-mCherry, Addgene) counterbalanced across sexes (male: *n* = 7 – 8/viral injection; female: *n* = 5 – 6/viral injection) into the middle / posterior PVT. Viral titer was a minimum of 4×10^12^ vg/mL. Mice were anesthetized in an induction chamber with 5% isoflurane in 2 L/min oxygen and then maintained under anesthesia through a nosecone with 1 – 2% isoflurane in 1 L/min oxygen. Warm saline (1 ml s.c., Baxter International Inc.) was injected to prevent dehydration, and bupivacaine (2 mg/kg s.c., Hospira Worldwide, Lake Forest, IL, USA) was injected into the scalp prior to incision. A 10 μL Nanofil syringe (World Precision Instruments, Sarasota, FL, USA) was lowered into the PVT (AP: −1.6, ML: −0.1, DV: −3.4, at a 6° angle) and the virus was injected at a rate of 50 nL/min. The syringe was then left in place for 10 minutes to allow for diffusion. Incisions were closed using wounds clips (Brain Tree Scientific Inc., Braintree, MA, USA), and lidocaine ointment and antibiotic ointment were applied to the incisions. Buprenorphine hydrochloride (0.03 mg/kg s.c., Reckitt & Colman Inc., Slough, UK) was administered for post-operative analgesia. Animals were handled and monitored during the week of recovery from surgery.

#### Immunohistochemistry.

Mice were deeply anesthetized and transcardially perfused as previously described [33]. Brains were then removed, postfixed, and frozen as described [33]. They were sliced coronally on a cryostat at 30 μm, and the sections were stored at −20°C in antifreeze solution (37.5% ethylene glycol, 20% sucrose in 0.03 M PBS). Every sixth section through the PVT was taken for processing and analysis, resulting in approximately 8 sections per brain. Tissue was processed as in [33], using the primary antibodies of polyclonal rabbit anti-PACAP-27/38 (1:300, Bioss Antibodies, Woburn, MA, USA; cat# BS0190R, lot# AI11065308), polyclonal guinea-pig anti-Cre (1:500, Synaptic Systems, Goettingen, Germany; cat# 257 004, lot# 3–17), and polyclonal sheep anti-c-Fos (1:1000, Osenses, Keswick, Australia, cat# OSC00007W, lot# YF3934611), and secondary antibodies of donkey anti-rabbit (Alexa Fluor^®^ 405) (1:200, Abcam, Cambridge, MA, USA; cat# ab175651, lot# GR3444080–1), donkey anti-guinea pig (Alexa Fluor^®^ 647) (1:200, Jackson ImmunoResearch Labs, West Grove, PA, USA, cat# 706-605-148, lot# 140553), and donkey anti-sheep (Alexa Fluor^®^ 488) (1:200, Abcam, Cambridge, MA, USA; cat# ab150177, lot# GR3441849–2). Pilot experiments were run to determine optimal staining conditions, and alternate sections did not show immunofluorescence when processed with the primary or secondary antibody omitted. All sections were processed at the same time under the same conditions. Sections were then mounted on slides and dried overnight in the dark, coverslipped with ProLong^®^ Diamond Antifade Mountant (Life Technologies, Carlsbad, CA, USA), and allowed to set for 24 hours before imaging. Imaging was conducted as previously reported [33]. Representative images were taken with a Leica DM6B Thunder episcope or a Leica SP8 VIS/405 HyVolution confocal microscope (Buffalo Grove, IL, USA). For analysis, all counting was performed manually, by an evaluator blind to the condition of the subject.

#### Histology.

Injection placement (Experiment 3) was confirmed by slide mounting and coverslipping alternate sections to those used for immunohistochemistry, and imaging them with a Leica DM5500 automated microscope. Images were captured with an Olympus DP71 high-resolution digital color camera (Waltham, MA, USA) with Slidebook V6 image acquisition and analysis software (3i, Denver, CO, USA). Representative images were taken with a Leica SP8 VIS/405 HyVolution confocal microscope. Three mice were removed from analysis from the Control group and five from the Gq group due to off-target injections (more than 0.3 mm lateral to the target region or into the anterior PVT) or no evidence of successful injection.

#### Data analysis.

Ensure, chow, and total kcal intake were analyzed as calories normalized to body weight in grams [(kcal / g BW) * 100]. Preference score was calculated by dividing the calories of Ensure consumed by total calories consumed (Ensure + chow) during the binge session and then multiplying by 100, for each animal per binge session ((2-hour Ensure kcal / 2-hour total kcal) * 100). Binge score was calculated by subtracting the average 2-hour chow intake of the male or female mice in the Control group from the Ensure intake of each male or female in the Binge group [(Individual subject Ensure intake) – (average control group 2-hour chow intake)]. Weight gain was calculated as percent change from body weight in Week 1. Analyses with non-access day intake lacked data from six females and two males from the Control group and two males and two females from the Binge group. Analyses also lacked data from two females and two males from the Binge group for 24-hr chow intake (24-hr chow and 24-hr kcal).

For Experiment 1, an independent *t*-test was used to compare the average of Ensure intake (kcal) to chow intake (kcal) across 2-hour sessions on access days, both in male and female Binge mice. A mixed ANOVA was used to analyze Ensure intake, 2-hr chow intake, 24-hr chow intake, total caloric intake, and body weight, with group and sex as between-subject measures and day (or week) of measurement as the within-subject measure. A repeated measures ANOVA was also used to examine binge score, with sex as the between-subject measure and day as the within-subject measure. For Experiment 2, a two-way ANOVA with group and sex as between-subjects measures was used examine levels of PACAP mRNA in the PVT. An independent *t*-test was used to compare males in the non-binge day group to females in the Control group. For Experiment 3, an independent *t*-test was used to compare immunohistochemical labeling between males and females, and between animals injected with CNO and those injected with saline. A mixed ANOVA, with virus as the between-subjects measure and drug injection as the within-subjects measure, was used to examine Ensure and chow intake on injection day. Significant main effects in all experiments were followed up with a Sidak pairwise comparison test. Sphericity was determined using Mauchly’s test, and a Greenhouse-Geisser correction was used when sphericity was violated. Significance was determined at *p* < 0.05. Schematic representations were created with BioRender.com.

## Results

### Experiment 1: Model of binge-type eating

In the Binge group, there was significantly greater consumption of Ensure than chow during the binge period (males: *t*(46) = −21.48, *p* < 0.001; females: *t*(46) = −28.16, *p* < 0.001). Males demonstrated an 87.8% preference and females demonstrated a 90.2% preference. Due to this high preference, further analyses of weekly intake in the Binge group during the 2-hour binge session examined only Ensure consumption.

**Figure 2.**
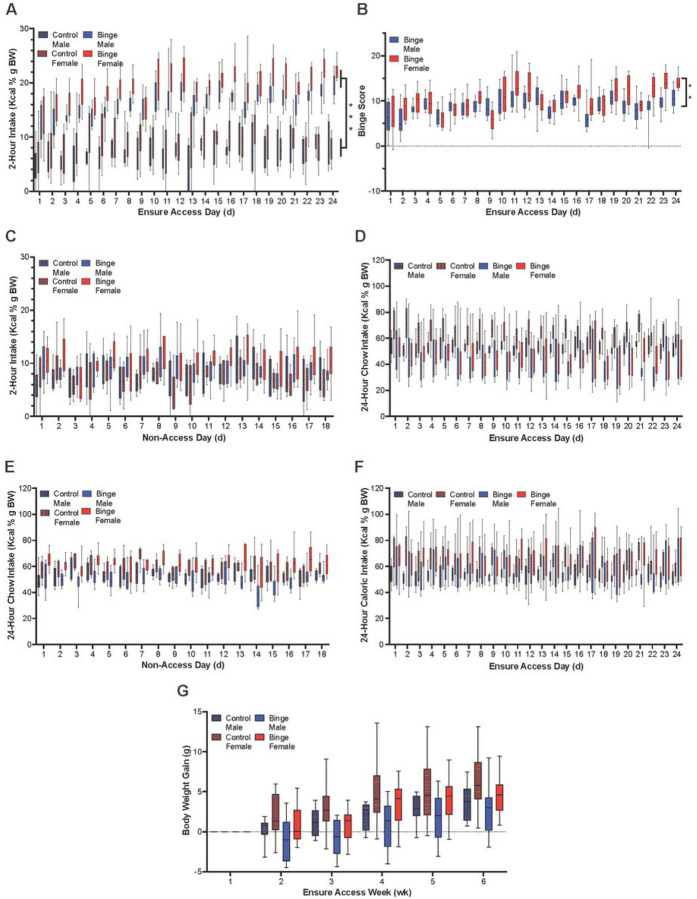
Model of binge-type eating (Experiment 1). **A.** For 2-hour intake on days with Ensure access, the Binge group consumed more calories per body weight than the Control group, with Binge intake increasing over the course of the experiment, and females in both the Binge and Control groups consuming more than males in the respective groups. **B.** Binge scores increased over the course of the experiment, and females had higher binge scores than males. **C.** For 2-hour chow intake on days without Ensure access, females in the Binge group consumed significantly more calories per body weight than females in the Control group. **D.** For 24-hour chow intake on days with Ensure access, the Binge group consumed fewer calories of chow per body weight than the Control group, and females in both the Binge and Control groups consumed more than males in the respective groups. **E.** For 24-hour chow intake on days without Ensure access, females consumed significantly more calories per body weight than males. **F.** For 24-hour calories consumed on days of Ensure access, females consumed significantly more total calories per body weight than males. **G.** Body weight increased over the course of the experiment, with females gaining more weight relative to their initial body weight than males. Box-and-whisker plots show minimum to maximum values, with a line at the mean. ***p < 0.001, **p < 0.01.

Examining 2-hour intake on days with Ensure access, comparison of Ensure consumption in the Binge group to chow intake at the same time in the Control group revealed a main effect of access day [*F*(11.16, 412.94 = 11.06, *p* < 0.001], group [*F*(1, 37) = 427.97, *p* < 0.001], and sex [*F*(1, 37) = 31.2, *p* < 0.001], as well as an interaction effect between access day and group [*F*(11.16, 412.94) = 4.03, *p* < 0.001]. Pairwise comparisons between days showed that both male and female mice in the Binge group increased their intake over the course of the experiment, having significantly higher intake of Ensure calories per body weight on the final day than on the first day (*p* < 0.001). In contrast, in the Control group, there was no difference in intake on the first day compared to the final day (*p* = 0.650). Mice in the Binge group consumed significantly more Ensure during the 2-hour session compared to mice in the Control group during the same 2-hour session (*p* < 0.001), and pairwise comparisons between sexes revealed that females compared to males in the Binge group consumed significantly more Ensure per body weight (*p* < 0.001), and females compared to males in the Control group consumed significantly more chow per body weight (*p* = 0.003) (Figure 2A). Examining binge scores also revealed a significant main effect of access day [*F*(7.66, 168.51) = 15.41, *p* < 0.001] and sex [*F*(1, 22) = 8.20, *p* = 0.009], as well an interaction effect between access day and sex [*F*(7.66, 168.51) = 3.64, *p* < 0.001]. Pairwise comparisons between days showed that binge score increased over the course of the experiment (*p* < 0.001). Females demonstrated significantly greater binge scores than males (*p* = 0.009) (Figure 2B). These results suggest that on days of access to Ensure, both male and female mice engage in binge-type eating, and that females binge to a greater extent than males.

Examining 2-hour intake on days without Ensure access also revealed a main effect of day [*F*(17, 493) = 2.97, *p* < 0.001)], but there was no discernable pattern of increase or decrease across time. There was a main effect of group [*F*(1, 29) = 9.72, *p* = 0.004], with the Binge group consuming significantly more calories of chow per body weight than the Control group (*p* = 0.004). There was no main effect of sex [*F*(1, 29) = 0.93, *p* = 0.342] and no interaction effect between sex and group [*F*(1, 29) = 1.53, *p* = 0.227]; however, comparisons of simple main effects revealed that, in females, the Binge group consumed significantly more per body weight during the 2-hour session on non-access days than the Control group (*p* = 0.004). This effect was not found in males (*p* = 0.197) (Figure 2C). These results suggest that, on days without Ensure access, Binge female but not Binge male mice engage in greater 2-hour intake than Controls.

Examining 24-hour chow intake on days with Ensure access revealed a main effect of access day [*F*(9.72, 389.15) = 2.16, *p* = 0.021]; however, there was again no discernable pattern of intake. There was also a main effect of group [*F*(1, 40) = 32.62, *p* < 0.001] and a main effect of sex [*F*(1, 40) = 7.88, *p* = 0.008], but no significant interaction effect between group and sex [*F*(1, 40) = 53.00, *p* = 0.091]. Females consumed more calories from chow per body weight than males (*p* = 0.008), and mice in the Binge group consumed significantly fewer calories from chow than mice in the Control group (*p* < 0.001) (Figure 2D).

Examining 24-hour chow intake on days without Ensure access, there was a main effect of day [*F*(17, 306) = 1.98, *p* = 0.012] and an interaction effect between day and group [*F*(17, 306) = 1.78, *p* = 0.030]; however, there was no discernable pattern of increase or decrease over time. There was again a main effect of sex [*F*(1, 18) = 5.43, *p* < 0.001], with females consuming significantly more calories per body weight from chow than males (*p* < 0.001). There was no main effect of group [*F*(1, 18) = 0.09, *p* = 0.767], although there was a significant interaction effect between group and sex [*F*(1, 18) = 5.43, *p* = 0.032]. Pairwise comparisons indicated that females in the Binge group consumed more chow than males in the Binge group (*p* < 0.001), and females in the Control group consumed more than males in the Control group (*p* = 0.028) (Figure 2E). These chow results together indicate that mice in the Binge group reduce their chow intake on days of Ensure access, but they consume the same amount as those in the Control group on days without Ensure access.

Examining 24-hour calories consumed (inclusive of both chow and Ensure, when relevant) on days of Ensure access, there was a main effect of sex [*F*(1, 40) = 12.13, *p* < 0.001], with females consuming significantly more total calories per body weight than males. There was no main effect of access day [*F*(9.85, 393.82) = 1.11, *p* = 0.324] and no main effect of group [*F*(1, 40) = 1.57, *p* = 0.218] (Figure 2F). These results suggest that, although mice in the Binge group consume significantly more calories during their binge sessions, this does not result in general overeating.

Examining change in bodyweight over the course of the experiment, there was a main effect of week [*F*(2.99, 119.92) = 42.26, *p* < 0.001], with weight increasing over the course of the experiment. There was a trend for a main effect of group [*F*(1, 40) = 3.63, *p* = 0.064] and a significant main effect of sex [*F*(1, 40) = 8.47, *p* = 0.006], but there was no interaction effect between group and sex [*F*(1, 40) = 0.011, *p* = 0.918]. The animals in the Control group showed a trend for more weight gain than animals in the Binge group (*p* = 0.064), and females gained more weight relative to their initial body weight than males (*p* = 0.006) (Figure 2G). These results suggest that, despite their greater consumption of calories in the 2-hour sessions, the Binge group did not gain significantly more weight than the Control group.

### Experiment 2: Gene expression of PACAP in the PVT relative to binge-type eating

**Figure 3.**
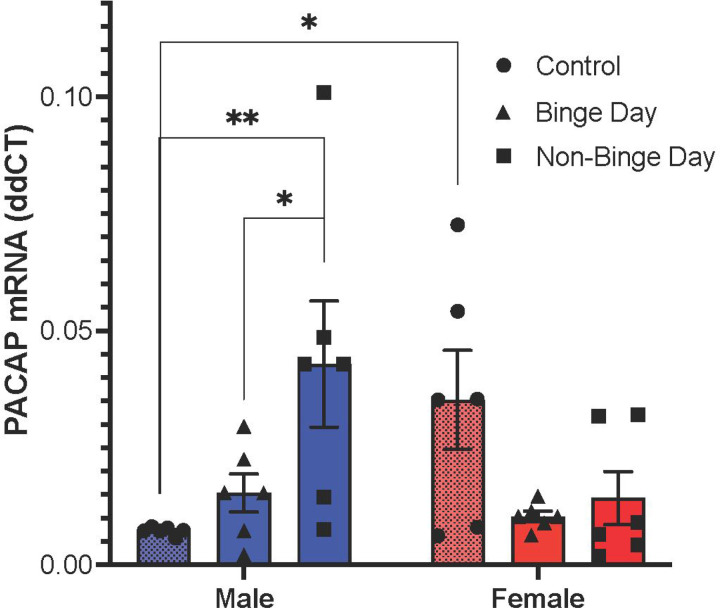
Gene expression of PACAP in the PVT relative to binge-type eating (Experiment 2). Between the Control groups, females had significantly higher levels of PACAP mRNA than males. Among males, the non-binge day group had significantly higher levels of PACAP mRNA than the Control group, but the binge day group had significantly lower levels of PACAP mRNA than the non-binge day group. Males in the non-binge day group had levels of PACAP mRNA that were statistically no different than Control females. Data are mean ± S.E.M. **p < 0.01, *p < 0.05.

Examining levels of PVT PACAP mRNA, results revealed no main effect of group [*F*(2, 30) = 2.16, *p* = 0.133] or sex [*F*(1, 30) = 0.09, *p* = 0.762], but there was a significant interaction effect between group and sex [*F*(2, 30) = 7.08, *p* = 0.003]. Pairwise comparisons revealed that Control females had significantly higher levels of PACAP than Control males (*p* = 0.014), but that non-binge day males had significantly higher levels than non-binge day females (*p* = 0.012), while there was no significant difference between the binge day groups (*p* = 0.641) (Figure 3). For males, pairwise comparisons indicated that the non-binge day group had significantly higher levels of PACAP than the Control group (*p* = 0.007) and that the binge day group had significantly lower levels of PACAP than the non-binge day group (*p* = 0.045). For females, pairwise comparisons showed a trend for lower levels of PACAP in the binge day group compared to the Control group (*p* = 0.078), and no significant differences between the non-binge day and Control group (*p* = 0.166) or the binge day and non-binge day group (*p* = 0.977). There was also no significant difference between levels of PACAP in the non-binge day males and Control females (*t*(10) = 0.44, *p* = 0.666). These results indicate that, in males but not females, a history of binge-type eating elevates levels of PACAP, and that these levels are decreased immediately prior to a binge eating session.

### Experiment 3. Effects of PACAP^+^ cell activity in the PVT on binge-type eating

**Figure 4.**
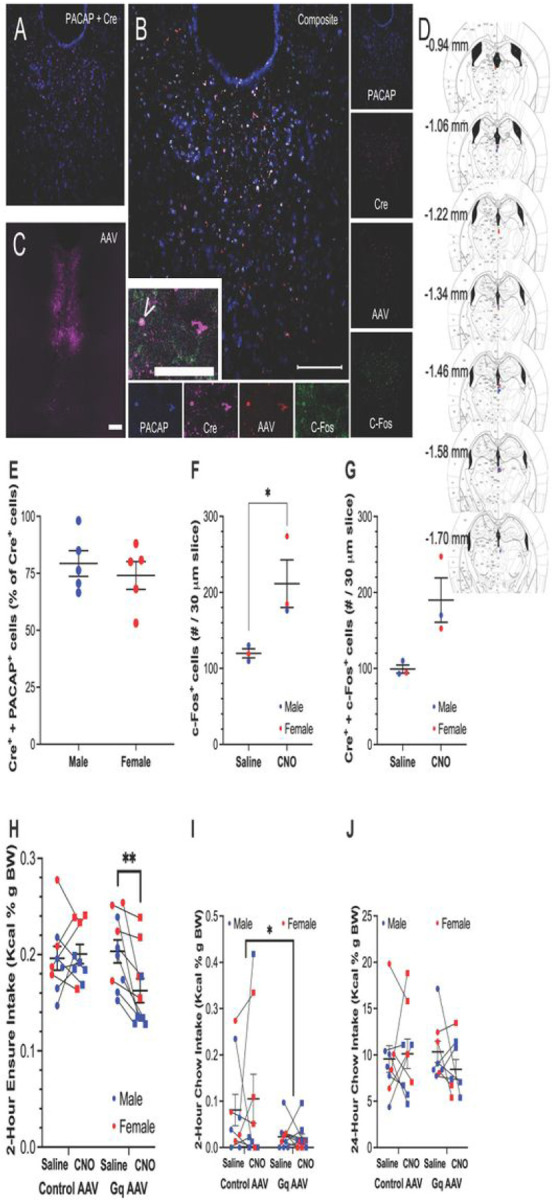
Effects of PACAP^+^ cell activity in the PVT of PACAP-Cre transgenic mice on binge-type eating (Experiment 3). **A.** Photomicrograph demonstrating co-labeling of PACAP (blue) and Cre (magenta) in the PVT of PACAP-Cre transgenic mice. **B.** Photomicrograph demonstrating co-labeling of PACAP (blue), Cre (magenta), the Cre-dependent excitatory DREADD (pAAV8-hSyn-DIO-hM3D(Gq)-mCherry) (red), and c-Fos (green) in the PVT of PACAP-Cre transgenic mice after systemic injection with CNO. Inset is higher-magnification image of main image, and shows a cell co-labeled for PACAP, Cre, and AAV (right) and a cell co-labeled for PACAP, Cre, AAV, and c-Fos (left, indicated by “V”). Scale bars = 200 μm. **C.** Injection track of the excitatory DREADD into the PVT. The AAV here is pseudo-colored magenta. Scale bar = 200 μm. **D.** Histological analysis found that virus injections were made between −0.94 and −1.70 mm posterior to bregma, in the middle and posterior subregions of the PVT. Dots indicate placement of on-target injections. Blue = male, red = female. **E.** In PACAP-Cre transgenic mice, a high percentage of Cre^+^ cells co-labeled with PACAP, with no significant difference between males and females. **F.** In mice that received the excitatory (G_q_) DREADD, systemic injection with CNO compared to saline resulted in a significant increase in c-Fos in the PVT. **G.** In mice that received the excitatory (G_q_) DREADD, systemic injection with CNO compared to saline increased c-Fos labeling in Cre^+^ neurons in the PVT. **H.** For effects of the excitatory (G_q_) DREADD on binge-type eating of Ensure, females overall consumed more Ensure than males, but injection of CNO resulted in reduced consumption of Ensure in the overall G_q_ group, due to a specific decrease in consumption in the males. **I.** For effects of the excitatory (G_q_) DREADD on chow intake during the 2-hour binge-type eating of Ensure, the overall G_q_ group ate less than the overall control AAV group. **J.** For effects of the excitatory (G_q_) DREADD on 24-hour chow intake on the day of Ensure access, there were no significant differences between groups. Data are mean ± S.E.M. **p < 0.01, *p < 0.05.

In PACAP-Cre transgenic mice, although there was more extensive labeling of Cre than of PACAP, 76% of all Cre^+^ cells co-labeled with PACAP, with no significant difference between males and females [*t*(8) = 0.64, *p* = 0.543] (Figures 4A and 4E). We believe that the PACAP antibody did not label all of the PACAP-containing cells in the PVT. In mice that received the excitatory (G_q_) DREADD in the PVT, systemic injection of CNO compared to saline resulted in a significant increase in c-Fos in the PVT [*t*(4) = 2.88, *p* = 0.045], and 64% compared to 33% of Cre^+^ neurons co-labeled with c-Fos (Figures 4B, 4F, and 4G). Histological analysis found that virus injections were made into the middle and posterior subregions of the PVT, between bregma −0.94 and −1.70 mm (Figure 4D). These results confirm that injection of CNO in PACAP-Cre transgenic mice that received the excitatory DREADD in the PVT resulted in excitation of PACAP^+^ cells in the PVT.

Examining effects of the excitatory (G_q_) DREADD on binge-type eating of Ensure, results showed that there was a significant main effect of sex [*F*(1, 15) = 10.87, *p* = 0.005] and a trend for a significant main effect of drug injection [*F*(1, 6) = 4.35, *p* = 0.054], and while there was no significant main effect of virus [*F*(1, 15) = 1.06, *p* = 0.319], there was a significant interaction effect between drug injection and virus [*F*(1, 15) = 7.03, *p* = 0.018] (Figure 4H). With females overall consuming more Ensure per body weight than males (*p* = 0.005), pairwise comparisons between drug injections revealed that, in the G_q_ group overall but not in the control AAV group overall, injection of CNO compared to saline resulted in significantly reduced consumption of Ensure (*p* = 0.004 and *p* = 0.701, respectively). Moreover, tests of simple main effects revealed that this decrease in Ensure intake following CNO in the G_q_ group was due to a significant decrease in Ensure intake in males (*p* = 0.004), but that females did not reach significance (*p* = 0.127) (Figure 4H). In contrast, when examining 2-hour chow intake during the same Ensure access sessions, there was no significant main effect of sex [*F*(1, 14) = 2.39, *p* = 0.144] or drug injection [*F*(1, 14) = 0.56, *p* = 0.468], although there was a significant main effect of virus [*F*(1, 14) = 5.01, *p* = 0.042], with the control AAV group overall eating more chow than the Gq group overall (Figure 4I). Examining 24-hour chow intake on the injection days, there was no significant main effect of sex [*F*(1, 13) = 4.04, *p* = 0.066], drug injection [*F*(1, 13) = 0.243, *p* = 0.630], or virus [*F*(1, 13) = 0.030, *p* = 0.865] (Figure 4J). These results suggest that enhancing the activity of PACAP^+^ cells in the PVT specifically decreases binge-type eating, and that this occurs in male but not female mice.

## Discussion

The first major result of our study is that limited access to Ensure (2 hours a day, 4 days per week) induces binge-type eating in male and female mice that meets the preclinical criteria for this behavior [4]. Moreover, this occurs to a greater extent in females compared to males. This sex-related difference aligns with previous rodent studies, which find that female compared to male rats are more likely to escalate to extreme progressive ratio self-administration of chocolate flavored sucrose pellets [17] and have higher demand for palatable foods at low cost [16], and that female compared to male C57Bl6/J mice escalate their intake more of palatable food when offered in daily, 2-hour sessions [13]. Despite their binge-type eating, our male and female mice did not consume significantly more total calories or gain more weight than our Control mice. This suggests that the binge model does not result in general overeating or excessive weight gain over six weeks of access. Indeed, previous studies of binge-type eating in rodents have noted that weight gain may only occur with sufficient duration of access to palatable food [6,9,11]. Thus, our model of binge-type eating in mice meets the preclinical criteria for binge eating and results in sex-related differences in behavior that are in line with prior findings.

A second major finding is that PACAP gene expression in the PVT is altered in relation to binge-type eating. Specifically, while females at baseline have higher PACAP levels than males, these levels increase in males, but not females, with a history of binge-type eating. This increased PACAP is then reduced immediately prior to a binge session, suggesting that it may endogenously disinhibit binge-type eating in males. The observed sex-related difference in baseline PACAP gene expression recapitulates our previous findings with PACAP gene expression and peptide levels in the PVT of rats and mice [33]. Notably, binge-type eating in males increases PACAP gene expression to levels comparable to those of binge-naïve females. This elevation in PACAP levels in males has previously been observed in another limbic region, the bed nucleus of the stria terminalis, following repeated or chronic exposure to abused drugs or stress [38–41]. Moreover, previous work has identified a sex-related difference in the role of PACAP in binge-type eating, with injection of PACAP-38 into the ventral tegmental area affecting males but not females [36]. In light of previous work from our lab, which found that temporary inhibition of the PVT decreases PACAP gene expression and increases sucrose seeking in male rats [30], we propose that, while female mice are initially more prone to binge eating behavior, perhaps in some way related to their elevated levels of PACAP, males can develop a similar proneness to binge eating as their baseline levels of PACAP increase following chronic or repeated exposure to limited-access palatable food.

A third major finding was that enhanced activity in middle / posterior PVT PACAP^+^ cells prior to the binge session attenuates binge-type eating, and this occurs in males more than in females. This reinforces the idea that a reduction in endogenous levels of PACAP in the PVT serves to disinhibit binge-type eating. In rats, temporary inhibition of the PVT with the GABA-A agonist, muscimol, has previously been found to promote chow intake [27], whereas photoactivation of the anterior PVT abolishes sucrose seeking during unexpected reward omission [26]. While these published findings are consistent with the results of our current study, they contrast with previous findings from our own group, which indicate that binge-like sucrose drinking is instead enhanced by neuropeptide-specific excitation of the posterior PVT in rats [23,24]. These discrepancies may be the result of differences in the binge paradigm, type of palatable food, or the specific population of PVT cells being manipulated. It has been hypothesized that disparate results from manipulations of the PVT may be due to variations in cell types and projection targets across the antero-posterior axis of the PVT [21,42]. Taken together, this suggests that the population of PACAP^+^ cells in the PVT exerts a specific influence on binge-type eating in male mice.

Overall, findings from the present experiments indicate that the PVT influences binge-type eating. Moreover, they suggest that changes in PVT PACAP expression related to binge eating are sex-dependent and occur following a history of binge eating as well as acutely prior to binge eating. They also indicate that PVT PACAP^+^ cell activity inhibits binge-type eating in male mice. Future studies are needed to identify the specific pathways through which this effect occurs. All together, the results suggest that PACAP should be further investigated as a possible pharmacotherapeutic target for the treatment of binge eating.
